# Book Notices

**Published:** 1870-07

**Authors:** 


### Book Notices.

Renal Diseases: A Clinical Guide to their Diagnosis and Treatment. By W. R. Basham, M.D.; Fellow of the Royal College of Physicians; Senior Physician to the Westminster Hospital, and Lecturer on Medicine, etc., etc. With Illustrations. Philadelphia: Henry C. Lea. 1870. For sale by S. C. Griggs & Co. Price $2.

This is a small-sized octavo volume of 304 pages, neatly printed and elegantly bound; and contains, in a small compass, a good summary of the present knowledge in the profession, in regard to renal diseases and their treatment.

Eighth Annual Report of the Board of Public Works, to the Common Council of the City of Chicago, for the year ending March 31st, 1869.

This is a volume of 207 pages, containing a very interesting statement of facts concerning the progress and present condition of the public improvements in this city. In regard to the extent of the water pipes, supplying water from the lake tunnel, the report says:—"There are now laid in the city, of mains of all sizes, 208 miles and 3117 feet. This extent of pipes laid exceeds that of any other city in our country, excepting New York, Brooklyn, and Philadelphia." Connected with the water pipes are 1070 fire hydrants. The total income received from water rents and taxes, during the year ending March 31st, 1869, amounted to $420,686.94." Total expenses for interest on debt and operating expenses $309,694.50; leaving a surplus revenue, to be applied to additional improvements, of $110,992.44. Total cost of the water works of the city, up to date of the report, $3,146,383.14. The sewers of the city are all laid on a definite plan or system, and the total length of sewers laid in the city, up to March 31st, 1869, was 110¼ miles." The report of the Board is accompanied by many interesting maps, and illustrations, and plans for future improvement.

